# A literature review and best practice advice for second and third trimester risk stratification, monitoring, and management of pre‐eclampsia: Compiled by the Pregnancy and Non‐Communicable Diseases Committee of FIGO (the International Federation of Gynecology and Obstetrics)

**DOI:** 10.1002/ijgo.13763

**Published:** 2021-07-29

**Authors:** Liona C. Poon, Laura A. Magee, Stefan Verlohren, Andrew Shennan, Peter von Dadelszen, Eyal Sheiner, Eran Hadar, Gerard Visser, Fabricio Da Silva Costa, Anil Kapur, Fionnuala McAuliffe, Amala Nazareth, Muna Tahlak, Anne B. Kihara, Hema Divakar, H. David McIntyre, Vincenzo Berghella, Huixia Yang, Roberto Romero, Kypros H. Nicolaides, Nir Melamed, Moshe Hod

**Affiliations:** ^1^ Department of Obstetrics and Gynecology The Chinese University of Hong Kong Hong Kong SAR China; ^2^ Department of Women and Children’s Health Faculty of Life Sciences and Medicine King’s College London London UK; ^3^ Department of Obstetrics Charité University Berlin Germany; ^4^ Department of Obstetrics and Gynecology B Soroka University Medical Center Ben‐Gurion University of the Negev Beersheba Israel; ^5^ Helen Schneider Hospital for Women Rabin Medical Center Petach Tikva Israel; ^6^ Sackler Faculty of Medicine Tel Aviv University Tel Aviv Israel; ^7^ Department of Obstetrics University Medical Center Utrecht The Netherlands; ^8^ Maternal Fetal Medicine Unit Gold Coast University Hospital and School of Medicine Griffith University Gold Coast Queensland Australia; ^9^ World Diabetes Foundation Bagsværd Denmark; ^10^ UCD Perinatal Research Centre School of Medicine University College Dublin National Maternity Hospital Dublin Ireland; ^11^ Jumeira Prime Healthcare Group Emirates Medical Association Dubai United Arab Emirates; ^12^ Latifa Hospital for Women and Children, Dubai Health Authority, Emirates Medical Association, Mohammed Bin Rashid University for Medica Sciences Dubai United Arab Emirates; ^13^ African Federation of Obstetricians and Gynaecologists Khartoum Sudan; ^14^ Divakar’s Speciality Hospital Bengaluru India; ^15^ University of Queensland Mater Clinical School Brisbane Queensland Australia; ^16^ Division of Maternal‐Fetal Medicine Thomas Jefferson University Philadelphia USA; ^17^ Department of Obstetrics and Gynecology Peking University First Hospital Beijing China; ^18^ Perinatology Research Branch, Division of Obstetrics and Maternal‐Fetal Medicine, Division of Intramural Research *Eunice Kennedy Shriver* National Institute of Child Health and Human Development, National Institutes of Health, US Department of Health and Human Services Bethesda, MD, and Detroit, MI USA; ^19^ Fetal Medicine Research Institute King’s College Hospital London UK; ^20^ Division of Maternal Fetal Medicine Department of Obstetrics and Gynecology Sunnybrook Health Sciences Centre University of Toronto Toronto ON Canada

**Keywords:** management, mean arterial pressure, monitoring, noncommunicable diseases, placental growth factor, prediction, pre‐eclampsia, risk stratification, second trimester, soluble fms‐like tyrosine kinase‐1, third trimester, timed delivery, uterine artery pulsatility index

## CONTENTS

**Table:** 

1. Executive summary	6
2. Target audience	8
3. Assessment of quality of evidence and grading of strength of recommendations	9
4. Pre‐eclampsia: Background	10
4.1. Introduction	10
4.2. Definition of pre‐eclampsia	10
4.3. Pathophysiology of pre‐eclampsia	11
5. Current method of monitoring for pre‐eclampsia	12
6. Risk stratification of pre‐eclampsia in the second and third trimesters of pregnancy	13
6.1. Short‐term prediction in women presenting with signs and symptoms of pre‐eclampsia	13
6.1.1. Placental growth factors	13
6.1.2. Soluble fms‐like tyrosine kinase 1 to placental growth factor ratio	13
6.2 Risk stratification and monitoring in asymptomatic high‐risk women	15
6.2.1. Antenatal maternal and fetal surveillance	15
7. Blood pressure, delivery, and postpartum management	16
7.1. Place of care	16
7.2. Antenatal maternal and fetal surveillance	16
7.3. Nonpharmacological therapy	17
7.4. Antihypertensive therapy	17
7.5. Magnesium sulfate and other strategies for women with pre‐eclampsia	18
7.6. Timed delivery	19
7.7. Postpartum care	21
8. Long‐term considerations associated with pre‐eclampsia	22
8.1 Cardiovascular disease	22
8.2 Early and late onset of pre‐eclampsia	22
8.3 Severity of pre‐eclampsia	23
8.4 Recurrence of pre‐eclampsia	23
8.5 End‐stage renal disease	23
8.6 Ophthalmic disease	23
9. Choice of automated blood pressure monitors	24
9.1 Blood pressure devices suitable for low‐resource settings	24
10. Cost‐effectiveness of supplementing current clinical practice with placental growth factor‐based tests	25
11. Considerations for universal aspirin in pre‐eclampsia prevention	26
12. Research priorities	27
13. References	27

## 1 EXECUTIVE SUMMARY

Pre‐eclampsia is a multisystem disorder that typically affects 2%–5% of pregnant women and is one of the leading causes of maternal and perinatal morbidity and mortality, especially when the condition is of early onset. Globally, 76 000 women and 500 000 babies die each year from this disorder. Furthermore, women in low‐resource countries are at a higher risk of developing hypertensive disorders of pregnancy and pre‐eclampsia compared with those in high‐resource countries. This is because socioeconomic, educational, and environmental disadvantages have historically beset vulnerable communities, leading to nutritional disparities, poor‐quality diet, obesity, and diabetes (before and during pregnancy), thus increasing the rates of pregnancy complications, in particular pre‐eclampsia.

Pre‐eclampsia has been traditionally defined as the onset of hypertension accompanied by significant proteinuria after 20 weeks of gestation. Recently, the definition of pre‐eclampsia has been broadened. Now the internationally agreed definition of pre‐eclampsia is that proposed by the International Society for the Study of Hypertension in Pregnancy (ISSHP).

According to ISSHP, pre‐eclampsia is defined as systolic blood pressure at ≥140 mmHg and/or diastolic blood pressure at ≥90 mmHg on at least two occasions measured 4 hours apart in previously normotensive women and is accompanied by ≥1 of the following new‐onset conditions at or after 20 weeks of gestation:

- Proteinuria: 24‐hour urine protein ≥300 mg/day; spot urine protein/creatinine ratio ≥30 mg/mmoL or ≥0.3 mg/mg, or urine dipstick testing ≥2+
- Other maternal organ dysfunction:
  ‐ Acute kidney injury (creatinine ≥90 µmol/L; >1.1 mg/dL);
  ‐ Liver involvement (such as elevated liver transaminases >40 IU/L) with or without right upper quadrant or epigastric pain;
  ‐ Neurological complications (including eclampsia, altered mental status, blindness, stroke, or more commonly hyperreflexia when accompanied by clonus, severe headaches, and persistent visual scotomata);
  ‐ Hematological complications (thrombocytopenia–platelet count <150 000/µL, disseminated intravascular coagulation, hemolysis);
  ‐ Uteroplacental dysfunction (such as fetal growth restriction, abnormal umbilical artery Doppler wave form or stillbirth).

Pre‐eclampsia can be subclassified into:

1. Early‐onset pre‐eclampsia (with delivery at <34^+0^ weeks of gestation).
2. Preterm pre‐eclampsia (with delivery at <37^+0^ weeks of gestation).
3. Late‐onset pre‐eclampsia (with delivery at ≥34^+0^ weeks of gestation).
4. Term pre‐eclampsia (with delivery at ≥37^+0^ weeks of gestation).

These subclassifications are not mutually exclusive. Early‐onset pre‐eclampsia is associated with a much higher risk of short‐ and long‐term maternal and perinatal morbidity and mortality. High‐quality evidence has demonstrated that early‐onset and preterm pre‐eclampsia can be effectively predicted by a Bayes‐based method‐derived model that incorporates maternal factors and a series of biological parameters measured at 11–13^+6^ weeks of gestation. When these high‐risk women (with estimated risk ≥1:100) are treated with 150 mg aspirin per night, from 11–14^+6^ weeks of gestation at a dose of approximately 150 mg to be taken every night until 36^+0^ weeks of gestation, the rates of early‐onset and preterm pre‐eclampsia can be reduced by 80% and 60%, respectively. FIGO (the International Federation of Gynecology and Obstetrics) endorsed this first‐trimester “screen and prevent” strategy for pre‐eclampsia and its pragmatic guidance was published in 2019.^1^

Current wider‐scale antenatal care is based on healthcare models developed in the early 20th century. In 1929 the UK Ministry of Health issued a Memorandum on Antenatal Clinics, recommending that women should first be seen at the 16th week of pregnancy and then at 24 and 28 weeks, fortnightly until 36 weeks, and then weekly until delivery. No explicit rationale was offered for the timing or clinical content of visits, yet these guidelines established the pattern of antenatal care that has been followed throughout the world to the present day.

A common assumption has prevailed that antenatal care should be concentrated around the third trimester of pregnancy, where most complications clinically materialize and adverse outcomes can be diagnosed. The current method of monitoring for pre‐eclampsia is based on this 90‐year‐old care pathway that requires that at every clinical visit, women are assessed for hypertension and proteinuria. However, even in the case of early‐onset disease, this approach detects hypertension and pre‐eclampsia only at a late stage of presentation, which does not necessarily allow optimization of care for both the mother and the fetus, namely stabilization of blood pressure, prophylactic corticosteroid for fetal lung maturation, and transferal to a tertiary referral unit prior to the need for immediate delivery, which is the only definitive treatment for this disorder.

In the past decade, major efforts have been made to develop tools for risk stratification and prediction of pre‐eclampsia in high‐risk women, as well as short‐term prediction in women presenting with signs and symptoms of pre‐eclampsia and those with confirmed pre‐eclampsia. FIGO brought together international experts to discuss and evaluate current knowledge on the topic and develop a document to frame the issues and suggest key actions to address the health burden posed by pre‐eclampsia.

FIGO’s objective, as outlined in this document, is: (1) to raise awareness of the links between pre‐eclampsia and poor maternal and perinatal outcomes as well as of the future health risks to mother and offspring, and demand a clearly defined agenda to tackle this issue globally; and (2) to create a consensus document, which provides guidance on prediction, risk stratification, monitoring, and management of pre‐eclampsia in the second and third trimester of pregnancy, and to disseminate and encourage its use.

Based on high‐quality evidence, the document outlines current global standards for the risk stratification, monitoring, and management of pre‐eclampsia in the second and third trimester of pregnancy.

It provides the most pragmatic advice for different resource settings—keeping in mind the feasibility, acceptability, and ease of implementation of the advice—to significantly lessen the health and economic burden caused by pre‐eclampsia. Suggestions are provided for a variety of different regional and resource settings based on their financial, human, and infrastructure resources, as well as for research priorities to bridge the current knowledge and evidence gap.

To address pre‐eclampsia, FIGO recommends the following:

**Public health focus:** there should be greater international attention focused on pre‐eclampsia and to the links between maternal health and noncommunicable diseases on the Sustainable Developmental Goals agenda. Public health measures to increase awareness, access, affordability, and acceptance of preconception counselling and antenatal and postnatal services for women of reproductive age should be prioritized. Greater efforts are required to raise awareness of the benefits of early antenatal visits targeted at women of reproductive age, particularly in low‐resource countries.

**Risk stratification and monitoring in asymptomatic women:** appropriate antenatal maternal and fetal surveillance should be put in place for high‐risk women for pre‐eclampsia. Where resources permit, the following could be included: guidance on recognition of symptoms and when to seek care; home blood pressure monitoring; regular formal clinical assessment (blood pressure measurement, dipstick proteinuria assessment and, where available, testing for hemoglobin, platelet count, serum creatinine, and liver transaminases); fetal ultrasonographic assessment of growth and umbilical artery Doppler; assessment of uterine artery Doppler.

**Management of women with confirmed pre‐eclampsia:** women with pre‐eclampsia should be assessed in hospital when first diagnosed. Thereafter, some women may be managed as outpatients once it is established that their condition is stable and they can be relied upon to monitor blood pressure at home and seek medical advice when there is rising/raised blood pressure. Appropriate antenatal maternal and fetal surveillance should be put in place. Where resources permit, the following could be included: maternal assessment by components of PIERS models (Pre‐eclampsia Integrated Estimate of Risk Scores), maternal laboratory testing, fetal ultrasonographic assessment of growth, umbilical artery Doppler, and fetal cardiotocography. At ≥32 weeks, if there is no access (or access is not yet possible) to fetal cardiotocography and ultrasound, the following should be used to assess fetal risk in hypertensive pregnancy: maternal age, symptoms, and dipstick proteinuria. For nonsevere hypertension management, elevated blood pressure should be treated with antihypertensive therapy with the target to achieve systolic blood pressure and diastolic blood pressure equal to or below 135 and 85 mmHg, respectively. Oral labetalol, nifedipine, and methyldopa should be considered as first‐line antihypertensive agents for nonsevere hypertension. Severe hypertension (systolic blood pressure ≥160 mmHg or diastolic blood pressure ≥110 mmHg) should be treated urgently with antihypertensive therapy in a monitored setting. Severely elevated diastolic blood pressure should be lowered to a target of 85 mmHg, but gradually over hours to days. Oral nifedipine, oral labetalol, intravenous labetalol, and intravenous hydralazine are considered as first‐line antihypertensive agents for severe hypertension. Magnesium sulfate is recommended for the prevention of eclampsia as well as a neuroprotective agent for the prevention of perinatal morbidity in preterm pre‐eclampsia requiring delivery at <32 weeks.

**Delivery plans for women with confirmed pre‐eclampsia:** delivery for pre‐eclampsia at any gestational age is recommended when there is one or more of the following conditions: abnormal neurological features such as severe intractable headache, repeated visual scotomata, eclampsia, or stroke; repeated episodes of severe hypertension despite maintenance treatment with three classes of antihypertensive agents; pulmonary edema or oxygen saturation <90%; progressive thrombocytopenia (particularly <50 × 10^9^/L or need for transfusion); abnormal and rising serum creatinine; abruption with evidence of maternal or fetal compromise; nonreassuring fetal status (including intrauterine fetal death). Mode of delivery is determined by several factors that include gestational age, fetal condition, and other concurrent obstetrics factors such as previous cesarean section.

**Postpartum care:** blood pressure should continue to be monitored after delivery until 6 days after birth, as it is likely to be highest 3–6 days after birth. Antihypertensive therapy that has been administered before birth should be continued after birth for as long as required to maintain blood pressure control. Consideration should be given to administering antihypertensive therapy for any hypertension diagnosed up to 6 days after delivery. Hypertensive pregnancy disorders should be acknowledged as predictors of long‐term maternal cardiovascular morbidity. The following measures should be implemented at 6–12 weeks after birth, and periodically thereafter, preferably yearly, following a pregnancy complicated by hypertensive disorders: history and physical examination, blood pressure measurements, and consideration of screening for other cardiovascular risk factors and for diabetes according to additional risk factors.

**Automated blood pressure devices:** Only automated blood pressure devices that have been shown to be accurate in pregnancy and pre‐eclampsia should be used.

## 2 TARGET AUDIENCE

This document is directed at multiple stakeholders with the intention of bringing attention to pre‐eclampsia, which is a common and potentially life‐threatening complication of pregnancy with grave consequences for both mothers and offspring. This document proposes to create a global framework for action for risk stratification, monitoring, and management of pre‐eclampsia.

The intended target audience includes:

**Healthcare providers:** all those qualified to care for pregnant women and their newborns but in particular those responsible for managing high‐risk women (obstetricians, maternal‐fetal medicine specialists, internists, pediatricians, neonatologists, general practitioners/family physicians, midwives, nurses, advance practice clinicians, nutritionists, pharmacists, community health workers, laboratory technicians, etc).

**Healthcare delivery organizations and providers:** governments, federal and state legislators, healthcare management organizations, health insurance organizations, international development agencies, and nongovernmental organizations.

**Professional organizations:** international, regional, and national professional organizations of obstetricians and gynecologists, internists, family practitioners, pediatricians, neonatologists, and worldwide national organizations dedicated to the care of pregnant women with pre‐eclampsia.

## 3 ASSESSMENT OF QUALITY OF EVIDENCE AND GRADING OF STRENGTH OF RECOMMENDATIONS

In assessing the quality of evidence and grading of strength of recommendations, this document follows the terminology proposed by the Grading of Recommendations, Assessment, Development and Evaluation (GRADE) working group.^2^ This system uses consistent language and graphical descriptions for the strength and quality of the recommendations and the evidence on which they are based. Strong recommendations are numbered as 1 and conditional (weak) recommendations are numbered 2 (Table 1). For the quality of evidence, cross‐filled circles are used: 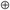 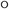 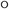 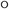 denotes very low‐quality evidence; 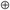 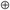 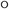 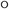 low quality; 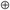 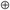 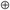 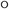 moderate quality; and 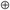 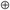 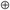 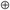 high quality evidence (Table 2).

**TABLE 1:** Interpretation of strong and conditional (weak) recommendations according to GRADE^a^, ^b^

Implications	1 = Strong recommendation phrased as “we recommend”	2 = Conditional (weak) recommendation phrased as “we suggest”
For patients	Nearly all patients in this situation would accept the recommended course of action. Formal decision aids are not needed to help patients make decisions consistent with their values and preferences	Most patients in this situation would accept the suggested course of action
For clinicians	According to the guidelines, performance of the recommended action could be used as a quality criterion or performance indicator	Decision aids may help patients make a management decision consistent with their values and preferences
For policy makers	The recommendation can be adapted as policy in most situations	Stakeholders need to discuss the suggestion

^a^ Adapted with permission of the American Thoracic Society. © 2021 American Thoracic Society. All rights reserved. Schunemann HJ, Jaeschke R, Cook DJ, et al. An official ATS statement: grading the quality of evidence and strength of recommendations in ATS guidelines and recommendations. *Am J Respir Crit Care Med* 2006;174:605–614. The *American Journal of Respiratory and Critical Care Medicine* is an official journal of the American Thoracic Society. Readers are encouraged to read the entire article for the correct context at: https://www.atsjournals.org/doi/full/10.1164/rccm.200602‐197ST. The authors, editors, and The American Thoracic Society are not responsible for errors or omissions in adaptations.

^b^ Both caregivers and care recipients need to be involved in the decision‐making process before adopting recommendations.

**TABLE 2:** Interpretation of quality of evidence levels according to GRADE^a^

Level of evidence	Definition
High 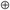 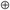 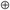 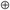	We are very confident that the true effect corresponds to that of the estimated effect
Moderate 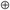 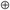 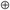 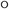	We are moderately confident in the estimated effect. The true effect is generally close to the estimated effect, but it may be slightly different
Low 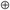 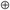 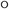 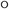	Our confidence in the estimated effect is limited. The true effect could be substantially different from the estimated effect
Very low 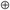 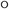 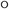 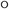	We have very little confidence in the estimated effect. The true effect is likely to be substantially different from the estimated effect

^a^ Adapted with permission from Balshem H, Helfand M, Schunemann HJ, Oxman AD, Kunz R, Brozek J, et al. GRADE guidelines: 3. Rating the quality of evidence. *J Clin Epidemiol*. 2011;64(4):401–406. © 2011 Elsevier.

## 4 PRE‐ECLAMPSIA: BACKGROUND

### 4.1 Introduction

Pre‐eclampsia is a multisystem disorder of pregnancy previously defined by the onset of hypertension accompanied by significant proteinuria after 20 weeks of gestation. Recently the definition of pre‐eclampsia has been broadened.^3^, ^4^, ^5^, ^6^ Pre‐eclampsia typically affects 2%–5% of pregnant women and is one of the leading causes of maternal and perinatal morbidity and mortality, especially when the condition is of early onset.^7^, ^8^ Globally, 76 000 women and 500 000 babies die each year from this disorder.^9^ Furthermore, women in low‐resource countries are at a higher risk of developing hypertensive disorders of pregnancy and pre‐eclampsia compared with those in high‐resource countries. This is because socioeconomic, educational, and environmental disadvantages have historically beset vulnerable communities leading to nutritional disparities, poor‐quality diet, obesity, and diabetes (before and during pregnancy), thus increasing the rates of pregnancy complications. FIGO (the International Federation of Gynecology and Obstetrics) has provided pragmatic guidance on addressing the management of hyperglycemia, nutrition, and obesity care during and after pregnancy.^10^, ^11^

### 4.2 Definition of pre‐eclampsia

Pre‐eclampsia is traditionally defined as development of hypertension and new proteinuria in a previously normotensive woman. The difficulty in interpreting epidemiological studies of pre‐eclampsia is due to the wide variation in the definitions of the disease. There are several definitions for the diagnosis of pre‐eclampsia, which have been reported in published literature and proposed by various professional bodies. Consequently, this has resulted in a number of different guidelines produced by professional bodies worldwide for the diagnosis and management of pre‐eclampsia.^3^, ^12^, ^13^, ^14^ An internationally agreed definition of pre‐eclampsia is, however, that of the International Society for the Study of Hypertension in Pregnancy (ISSHP)^6^ (Box 1), which is endorsed by FIGO.

Box 1 **Diagnosis of hypertensive disorders in pregnancy according to the Society for the Study of Hypertension in Pregnancy^6^**

**Gestational hypertension**:

- Persistent de novo hypertension (systolic blood pressure ≥140 mmHg and/or diastolic blood pressure ≥90 mmHg) after 20 weeks of gestation in the absence of features of pre‐eclampsia.

**Pre‐eclampsia de novo**:

- Gestational hypertension accompanied by ≥1 of the following new‐onset conditions at or after 20 weeks of gestation:
  ‐ Proteinuria: 24‐hour urine protein ≥300 mg/day; spot urine protein/creatinine ratio ≥30 mg/mmoL or ≥0.30 mg/mg, or urine dipstick testing ≥2+
  ‐ Other maternal organ dysfunction:
    ▪ Acute kidney injury (creatinine ≥90 µmol/L; >1.1 mg/dL);
    ▪ Liver involvement (such as elevated alanine aminotransferase (ALT) or aspartate transaminase (AST) >40 IU/L with or without right upper quadrant or epigastric pain);
    ▪ Neurological complications (including eclampsia, altered mental status, blindness, stroke, or more commonly hyperreflexia when accompanied by clonus, severe headaches, and persistent visual scotomata);
    ▪ Hematological complications (thrombocytopenia–platelet count <150 000/µL, disseminated intravascular coagulation, hemolysis);
    ▪ Uteroplacental dysfunction (such as fetal growth restriction, abnormal umbilical artery Doppler wave form or stillbirth).

**Superimposed pre‐eclampsia on chronic hypertension:**

- Women with chronic essential hypertension develop any of the above maternal organ dysfunctions consistent with pre‐eclampsia.
- Increase in blood pressure per se is not sufficient to diagnose superimposed pre‐eclampsia.
- In the absence of pre‐existing proteinuria, new‐onset proteinuria in the setting of a rise in blood pressure is sufficient to diagnose superimposed pre‐eclampsia.

In women with proteinuric renal disease, an increase in proteinuria during pregnancy is not sufficient per se to diagnose superimposed pre‐eclampsia.

Refer to Section 7.4 “Antihypertensive therapy” for the definition of severe hypertension.

As described in the FIGO initiative on pre‐eclampsia, published in 2019,^1^ according to the associated risks of maternal and perinatal morbidity and mortality, pre‐eclampsia can be further subclassified into:

1. Early‐onset pre‐eclampsia (with delivery at <34^+0^ weeks of gestation).
2. Preterm pre‐eclampsia (with delivery at <37^+0^ weeks of gestation).
3. Late‐onset pre‐eclampsia (with delivery at ≥34^+0^ weeks of gestation).
4. Term pre‐eclampsia (with delivery at ≥37^+0^ weeks of gestation).

These subclassifications are not mutually exclusive. High‐quality evidence has demonstrated that early‐onset and preterm pre‐eclampsia can be effectively predicted by a Bayes‐based method‐derived model that incorporates maternal factors and a series of biological parameters measured at 11–13^+6^ weeks of gestation.^15^ When these high‐risk women (with estimated risk ≥1:100) are treated with 150 mg aspirin per night, from 11–14^+6^ weeks of gestation until 36^+0^ weeks of gestation, the rates of early‐onset and preterm pre‐eclampsia can be reduced by 80% and 60%, respectively.^16^ FIGO has endorsed this first‐trimester “screen and prevent” strategy for pre‐eclampsia and its pragmatic guidance was published in 2019.^1^ In the present guidance, we focus on the risk stratification, monitoring, and management of pre‐eclampsia in the second and third trimester of pregnancy.

### 4.3 Pathophysiology of pre‐eclampsia

Pre‐eclampsia is a heterogeneous, multifactorial syndrome and its etiology is far from understood. Details on the different etiological hypotheses are beyond the scope of this best practice advice. Specific reviews can be found elsewhere.^17^, ^18^, ^19^ However, important understanding of the pathophysiology of the disease has been gained by the discovery of the disturbed angiogenic and antiangiogenic balance in women destined to develop pre‐eclampsia and associated adverse events. Women with pre‐eclampsia exhibit high circulating serum levels of fms‐like tyrosine kinase 1 (sFlt‐1) and low levels of placental growth factor (PLGF).^20^ Experimentally, iatrogenic overexpression of sFlt‐1 in pregnant rats leads to hypertension, proteinuria, and glomerular endotheliosis—a histological hallmark of pre‐eclampsia. In a baboon model for pre‐eclampsia (uterine ligation), restoring the angiogenic balance by application of recombinant human PLGF (rhPLGF) ameliorated pre‐eclampsia symptoms, such as hypertension and proteinuria.^21^ Application of short interfering RNAs (siRNAs) leads to reduction of blood pressure and proteinuria via silencing of sFlt‐1 expression in experimental models (primates and mice). In humans, extracorporeal removal of excessively elevated sFlt‐1 in women with early‐onset pre‐eclampsia led to a prolongation of the disease.^22^, ^23^ These lines of evidence highlight the concept of a disturbed angiogenic balance as being central to the pathophysiology of the disease. This has led to the development of sFlt‐1 and PLGF as markers for diagnosis, prognostication, and prediction of the disease, as discussed below.

## 5 CURRENT METHOD OF MONITORING FOR PRE‐ECLAMPSIA

Current wider‐scale antenatal care is based on healthcare models developed in the early 20th century. In 1929 the UK Ministry of Health issued a Memorandum on Antenatal Clinics, recommending that women should first be seen at the 16th week of pregnancy and then at 24 and 28 weeks, fortnightly until 36 weeks, and then weekly until delivery.^24^ No explicit rationale was offered for the timing or clinical content of visits, yet these guidelines established the pattern of antenatal care that has been followed throughout the world to the present day.

A common assumption has prevailed that antenatal care should be concentrated around the third trimester of pregnancy, where most complications clinically materialize and adverse outcomes can be diagnosed. The current method of monitoring for pre‐eclampsia is based on this 90‐year‐old care pathway that requires that at every clinical visit, women are assessed for hypertension and proteinuria. However, even in the case of early‐onset disease, this approach detects hypertension and pre‐eclampsia only at a late stage of presentation, which does not allow optimization of care for both the mother and the fetus, namely stabilization of blood pressure, prophylactic corticosteroid for fetal lung maturation, and transfer to a tertiary referral unit prior to the need for immediate delivery, which is the only definitive treatment for this disorder.

In the past decade, major efforts have been made to develop tools for risk stratification and prediction of pre‐eclampsia in high‐risk women, as well as short‐term prediction in women presenting with signs and symptoms of pre‐eclampsia. An overview of the existing literature is summarized in the following section.

## 6 RISK STRATIFICATION OF PRE‐ECLAMPSIA IN THE SECOND AND THIRD TRIMESTERS OF PREGNANCY

### 6.1 Short‐term prediction in women presenting with signs and symptoms of pre‐eclampsia

#### 6.1.1 Placental growth factor

In addition to their use as a first‐trimester screening tool, PLGF‐based tests have been shown to have high diagnostic accuracy in women with suspected pre‐eclampsia. A recent prospective multicenter study demonstrated that low circulating maternal PLGF concentrations had high sensitivity (96%; 95% CI, 89–99) and negative predictive value (98%; 95% CI, 93.0–99.5) in diagnosing pre‐eclampsia that required delivery within 14 days in women who presented with suspected pre‐eclampsia.^25^

This UK PELICAN study^25^ showed that the Triage PLGF test at a cutoff of 100 pg/mL (with ≥100 pg/mL considered a normal result) had a negative predictive value of 98% when used to rule out pre‐eclampsia that needed delivery within the next 14 days. Ruling in women with an abnormal result of less than 12 pg/mL (the lower limit of detection) yielded high specificity (>90%) for the same endpoint. These tests were valid in women presenting with suspected pre‐eclampsia, which includes women with hypertension, proteinuria, fetal growth restriction, or symptoms suggestive of pre‐eclampsia such as headaches or epigastric pain. The test works well between 20 and 34^+6^ weeks of gestation. The test has some value after 35 weeks (up to 37 weeks) but is not as good.^25^

The authors went on to implement these thresholds in a pragmatic stepped‐wedge trial to see if knowledge of the test influenced behaviors and outcomes. The PARROT trial demonstrated that at an average of 32 weeks of gestation, the availability of PLGF results (using the Triage PLGF test) substantially reduced the time to clinical confirmation of pre‐eclampsia (1.9 vs 4.1 days; time ratio 0.36; 95% CI, 0.15–0.87; *P* = 0.027) and reduced adverse maternal outcomes (4% vs 5%; adjusted odds ratio 0.32, 95% CI, 0.11–0.96; *P* = 0.043), supporting the adoption of PLGF‐based testing into routine clinical practice.^26^

The high negative predictive value of PLGF‐based tests supports their use as a “rule out” tool in women with suspected disease preterm. We suggest their use alongside clinical assessment to help rule out pre‐eclampsia in women suspected of developing the disease. While angiogenic markers may be of value in pre‐eclampsia given the number of women with both hypertension and dipstick proteinuria at baseline, this remains to be established. In addition, further work is needed to establish the value of repeated PLGF measurements in women presenting with suspected or confirmed pre‐eclampsia, particularly after 35 weeks.

It is important to mention that currently there are four PLGF‐based tests commercially available. Furthermore, that PLGF has different isoforms. The specific rule in/out criteria are dependent on the exact assay (which have different detection characteristics depending on which isoform of PLGF is detected), and whether a ratio of sFlt‐1 to PLGF is used. In addition, the prevalence of pre‐eclampsia, or the endpoint used, is variable in the different clinical studies using different assays, making direct comparison between studies difficult as the predictive values are highly dependent on prevalence in the given setting.

The COMPARE study^27^ evaluated three of these assays in the same population of women, using the manufacturer's recommended cutoffs: Triage PLGF test (Quidel Corporation, San Diego, CA, USA), the DELFIA‐Xpress PLGF 1‐2‐3‐test (PerkinElmer Inc., Waltham, MA, USA), and the Elecsys immunoassay sFlt‐1/PLGF ratio (Roche Diagnostics, Mannheim, Germany) (Table 3). Similar performance was demonstrated in the prediction of need for delivery within 14 days in women with suspected pre‐eclampsia.

**TABLE 3:** Rule‐in and rule‐out thresholds of commercially available assays

	Triage PLGF test	Elecsys sFlt‐1/PLGF ratio	DELFIA Xpress PLGF 1‐2‐3 test	BRAHMS sFlt‐1/PLGF plus ratio
Recommended rule‐out threshold	≥100 pg/mL	≤38	≥150 pg/mL	>55
Suggested rule‐in threshold	<12 pg/mL	>85	<50 pg/mL	>188
Relevant study	PELICAN^25^ PARROT^26^	PROGNOSIS^28^ INSPIRE^29^	COMPARE^27^	Cheng et al.^30^

The ultimate choice of which assay to use will depend on cost, availability, and clinical utility such as ease of use. All current tests appear to be valuable. The Triage PLGF test and the Elecsys immunoassay sFlt‐1/PLGF ratio have been recommended by the National Institute for Health and Care Excellence (NICE) as a rule‐out test for pre‐eclampsia at less than 35 weeks.^31^ The National Health Service (NHS) England has funded initiatives to roll out these tests nationally for suspected pre‐eclampsia at less than 35 weeks.

#### 6.1.2 Soluble fms‐like tyrosine kinase 1 to placental growth factor ratio

The role of the sFlt‐1/PLGF ratio to predict adverse outcomes related to pre‐eclampsia was investigated in a prospective study with 616 women presenting with signs and symptoms of the disease.^32^ Women were eligible for enrolment when they presented with either elevated blood pressure or proteinuria and/or symptoms such as headache, visual symptoms, right upper quadrant pain, or edema. The primary endpoint was the development of maternal and/or fetal adverse events related to pre‐eclampsia within 2 weeks. Maternal adverse events were defined as a combination of hypertension and abnormal liver function tests, disseminated intravascular coagulation, pulmonary edema, or eclampsia. Fetal adverse events included indicated delivery, fetal growth restriction, or fetal or neonatal death. Adverse events occurred in 43.5% of all patients (*n* = 268) and in 33.5% of women presenting at less than 34 weeks of gestation (*n* = 59). Women who had an adverse event related to pre‐eclampsia had a significantly elevated sFlt‐1/PLGF ratio compared with those who did not (47.0, interquartile range [IQR] 15.5–112.2 vs 10.8, IQR 4.1–28.6; *P* < 0.001). In women who presented at less than 34 weeks of gestation (*n* = 176), the results were more striking (226.6, IQR 50.4–547.3 vs 4.5, IQR 2.0–13.5; *P* < 0.001). For women who presented before 34 weeks of gestation, the addition of the sFlt‐1/PLGF ratio to hypertension and proteinuria significantly improved the prediction for subsequent adverse outcomes (area under the receiver operating characteristic curve (AUC) 0.93 for hypertension, proteinuria, and sFlt‐1/PLGF ratio versus 0.84 for hypertension and proteinuria alone; *P* < 0.001). Delivery occurred within 2 weeks of presentation in 86.0% of women with an sFlt‐1/PLGF ratio greater than 85 compared with 15.8% of women with an sFlt‐1/PLGF ratio less than 85 (hazard ratio, 15.2; 95% CI, 8.0–28.7).^32^

In the PROGNOSIS study,^28^ a prospective observational study conducted in 14 countries, the ability of the sFlt‐1/PLGF ratio to predict the absence of pre‐eclampsia within 1 week and to predict the presence of pre‐eclampsia within 4 weeks in women with signs and symptoms of pre‐eclampsia was investigated. This study included 1050 pregnant women aged 18 years or older at 24–36^+6^ weeks of gestation with clinical symptoms of the disease such as new onset of hypertension or aggravation of pre‐existing hypertension; new onset of proteinuria or aggravation of existing proteinuria; the presence of typical symptoms of the disease such as headache, right upper quadrant abdominal pain, edema, or weight gain; as well as an abnormal uterine artery Doppler. The prevalence of pre‐eclampsia in the full dataset was 17.8%. In the development cohort of 500 women, the single cutoff of 38 was found to be predictive for the primary endpoint, which was then evaluated in the validation cohort of another 550 women. In women with suspected pre‐eclampsia according to the PROGNOSIS criteria, the negative predictive value of an sFlt‐1/PLGF ratio ≤38 for ruling out the occurrence of pre‐eclampsia within 1 week was 99.3% (95% CI, 97.9–99.9). The positive predictive value of an sFlt‐1/PLGF ratio >38 for ruling in the occurrence of pre‐eclampsia within 4 weeks was 36.7% (95% CI, 28.4–45.7). The positive predictive value for the occurrence of a combined endpoint of pre‐eclampsia/eclampsia/HELLP syndrome (hemolysis, elevated liver enzymes, and low platelet count) or maternal and/or fetal adverse outcomes within 4 weeks was 65.5% (95% CI, 56.3–74.0).^28^

In an exploratory post hoc analysis of the PROGNOSIS dataset it was demonstrated that an sFlt‐1/PLGF ratio of ≤38 can rule out pre‐eclampsia within 4 weeks with a negative predictive value of 94.3% (95% CI, 91.7–96.3).^33^ Evidence from this analysis shows the importance of repeated measurements in women with signs and symptoms of the disease. Women with suspected pre‐eclampsia who developed the disorder had a significantly larger median increase in the sFlt‐1/PLGF ratio at 2 weeks (delta [Δ] 31.22) and 3 weeks (Δ48.97) after the first blood draw, compared with those who did not (Δ1.45 and Δ2.39, respectively; *P* < 0.001).

These results were validated in the PROGNOSIS Asia study.^34^ This multicenter study enrolled 764 women with suspected pre‐eclampsia in 25 centers in Asia. Suspected pre‐eclampsia was defined as in the PROGNOSIS study; however, only severe persistent epigastric pain and new onset of visual disturbances were considered as potential symptoms related to pre‐eclampsia. In this study an sFlt‐1/PLGF ratio cutoff of ≤38 was shown to have a negative predictive value of 98.6% (95% CI, 97.2–99.4) for ruling out pre‐eclampsia within 1 week and a ratio >38 demonstrated a positive predictive value of 30.3% (95% CI, 23.0–38.5) for ruling in pre‐eclampsia within 4 weeks. The positive predictive value for the occurrence of a combined endpoint of pre‐eclampsia/eclampsia/HELLP syndrome or maternal and/or fetal adverse outcomes within 4 weeks was 65.0% (95% CI, 56.6–72.8).^34^

A prospective cohort study of nulliparous women investigated the added value of the sFlt‐1/PLGF ratio in a high‐risk and low‐risk population.^35^ High‐risk of pre‐eclampsia was defined as either: (1) maternal characteristics, using the UK NICE guideline; or (2) elevated 20 weeks uterine artery Doppler, defined as a mean pulsatility index in the highest decile. Blood sampling was performed at approximately 20, 28, and 36 weeks. The primary outcomes were pre‐eclampsia and delivery <28 weeks or pre‐eclampsia and delivery <37 weeks (for 20 weeks sample); pre‐eclampsia and delivery <37 weeks (28 weeks sample); and pre‐eclampsia with severe features (36 weeks sample). A total of 4099 women were recruited, the incidence of pre‐eclampsia was 6.5% (265/4099) in total, 0.1% before 28 weeks, 0.65% before 36 weeks, and 2.8% developed severe pre‐eclampsia after 36 weeks. The screening performance at 20, 28, and 36 weeks was 0.70 (95% CI, 0.43–0.97), 0.80 (95% CI, 0.70–0.89), and 0.81 (95% CI, 0.77–0.86), respectively. Women with an sFlt‐1/PLGF ratio >38 (*n* = 19) at 28 weeks had an incidence of pre‐eclampsia of 32% leading to preterm delivery. The positive predictive value was similar in low‐ and high‐risk women (33% vs 31%, *P* = 0.91). At 36 weeks, women with an sFlt‐1/PLGF ratio >38 (*n* = 566) had an incidence of severe pre‐eclampsia of 10%. Among women with no prior risk factors, an sFlt‐1/PLGF ratio ≤38 had a high negative predictive value for subsequent development of severe disease (>99%). Sovio et al.^35^ tested the cutoffs of 85 (<34 weeks) and 110 (>34 weeks) in their cohort. Four out of seven women with an sFlt‐1/PLGF ratio >85 at 28 weeks delivered preterm with a diagnosis of pre‐eclampsia (positive predictive value 57%). At 36 weeks, 70 women had an sFlt‐1/PLGF ratio >110 and 21 developed severe disease (positive predictive value 30%). The positive predictive value was similar comparing women with and without prior risk factors (36% and 24%, respectively).^35^

### 6.2 Risk stratification and monitoring in asymptomatic high‐risk women

#### 6.2.1 Antenatal maternal and fetal surveillance

**Table:** 

Best practice advice^a^	Quality of evidence	Strength of recommendation
Pregnant women who screen positive as high risk for pre‐eclampsia and the related placental disorders of gestational hypertension, fetal growth restriction, and stillbirth should be offered increased antenatal maternal and fetal surveillance.	Low 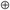 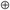 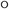 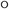	Strong
In high‐risk women, antenatal maternal surveillance should include guidance on recognition of symptoms (e.g. headache, visual disturbances, chest pain, dyspnea, epigastric pain, right upper quadrant pain, or vaginal bleeding) and when to seek care.	Low 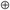 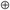 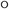 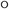	Strong
In high‐risk women, antenatal surveillance should include daily home blood pressure monitoring, where resources permit.	Low 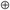 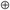 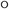 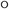	Conditional
If possible, high‐risk women should be assessed by the formal health system at least once every 2 weeks until 27^+6^ weeks and weekly thereafter; such assessments should include symptom screening, blood pressure measurement, dipstick proteinuria assessment (if women are hypertensive) and, where available, hemoglobin, platelet count, serum creatinine, and serum aspartate transaminase (AST) or alanine aminotransferase (ALT) tests.	Low 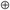 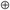 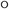 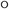	Conditional
In high‐risk women, fetal surveillance should include fetal biometry, amniotic fluid assessment, and umbilical artery Doppler, at least every 2–4 weekly where resources permit. Should evidence of decreased fetal growth velocity become evident, both maternal and fetal surveillance should be increased to at least weekly assessments, even if the woman remains normotensive and asymptomatic.	Low 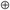 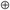 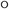 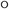	Conditional
Where there is either limited or no access to ultrasound, serial symphysis–fundal height measurements should be performed at least every 2 weeks during the care of high‐risk women by appropriately trained care providers (preferably the same each time).	Low 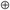 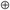 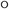 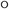	Strong

^a^ “High risk” for the first trimester is defined according to Poon et al.^1^ Otherwise, high risk is defined by the ISSHP criteria.^6^

The Edinburgh antenatal care visit paradigm was developed in large part to assist in screening for and diagnosing pregnancy hypertension. The introduction of that paradigm of 4‐weekly visits from booking until 27^+6^ weeks, fortnightly visits from 28^+0^–35^+6^ weeks, and weekly visits from 36^+0^ weeks until delivery was associated with accelerated improvements in maternal survival. The World Health Organization (WHO) focused antenatal care model (four visits per pregnancy) was associated with less optimal perinatal outcomes compared with the Edinburgh paradigm^36^; hence the introduction of the eight‐encounter model in 2017.^37^ Both blood pressure measurement and proteinuria screening are integral elements of a WHO‐compliant antenatal visit program; however, the inclusion of regular proteinuria assessment at all visits did not follow formal evidence review. Canada has undertaken such a review, and the national advice now specifically states that proteinuria screening should not be performed as part of routine antenatal care.^38^

## 7 BLOOD PRESSURE, DELIVERY, AND POSTPARTUM MANAGEMENT

### 7.1 Place of care

**Table:** 

Pragmatic practice advice	Quality of evidence	Strength of recommendation
We recommend that women with pre‐eclampsia should be assessed in hospital when first diagnosed. Thereafter, some women may be managed as outpatients once it is established that their condition is stable, and they can be relied upon to monitor blood pressure at home and seek medical advice when there is rising/raised blood pressure.	Low 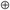 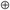 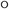 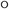	Strong

The level of blood pressure itself is not a reliable way to stratify immediate risk in pre‐eclampsia because some women may develop serious organ dysfunction at relatively mild levels of hypertension.

### 7.2 Antenatal maternal and fetal surveillance

**Table:** 

Best practice advice	Quality of evidence	Strength of recommendation
*Maternal surveillance*		
We recommend that beyond blood pressure and proteinuria measurement, maternal assessment of women with gestational hypertension, with or without proteinuria, should include components of PIERS models (Pre‐eclampsia Integrated Estimate of Risk Scores).	Moderate 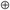 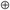 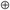 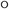	Conditional
We recommend that maternal laboratory testing should occur, at minimum, twice weekly for inpatients.	Low 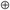 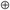 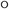 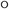	Conditional
We suggest that maternal laboratory testing should occur weekly for outpatients.	Low 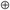 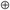 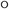 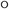	Conditional
*Fetal surveillance*		
We recommend that where available, ultrasound be performed once every 2 weeks to assess fetal growth, and at least once every 2 weeks to assess liquor volume and umbilical artery Doppler.	Low 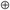 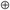 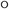 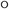	Conditional
We recommend fetal cardiotocography (CTG) to monitor the fetal condition. In early fetal growth restriction before 34 weeks, CTG should be performed daily. Preferably by using computerized CTG to assess fetal heart rate variation.	Low 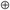 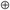 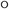 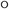 Low 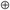 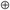 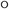 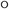	Strong Conditional
We recommend at <34 weeks when there is fetal growth restriction, and where trained personnel are available to perform and interpret the assessment, Doppler velocimetry of the ductus venosus be performed, to assess the risk of adverse perinatal outcome.	Low 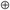 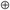 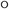 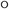	Conditional
We recommend against the use of the biophysical profile to monitor growth restricted fetuses at risk in hypertensive pregnancy.	Low 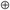 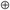 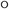 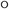	Conditional
We suggest that at ≥32 weeks, if there is no access (or access is not yet possible) to fetal CTG and ultrasound, the following should be used to assess fetal risk in hypertensive pregnancy: maternal age, symptoms, and dipstick proteinuria.	Low 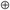 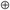 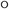 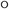	Conditional

Beyond assessment of blood pressure and proteinuria, maternal assessment should include the components of the fullPIERS models (https://pre‐empt.obgyn.ubc.ca/evidence/fullpiers) that are predictive of adverse maternal outcome in hypertensive pregnancy and pre‐eclampsia, specifically, when performed at least twice weekly.^39^, ^40^, ^41^ The models incorporate gestational age but are not restricted to a specific gestational age range, like the PREP model developed for use in pre‐eclampsia before 34 weeks.^42^ Without ready access to laboratory results, the miniPIERS model includes systolic blood pressure, dipstick proteinuria, parity, gestational age, and symptoms (headache/visual symptoms, chest pain/dyspnea, abdominal pain with vaginal bleeding); model performance is improved with the addition of pulse oximetry.^39^ With ready access to laboratory results, fullPIERS includes gestational age, chest pain/dyspnea, pulse oximetry, platelet count, serum creatinine, and aspartate transaminase (AST) or alanine aminotransferase (ALT).^40^ While clonus reflects central nervous system irritability, the reproducibility of clonus testing (in the maternity setting) and its independent predictive value for adverse outcome is uncertain.

The fetuses of women with hypertension are at increased risk of mortality and morbidity. While multiple methods are available to monitor the fetuses of hypertensive pregnancies, no strategy of various methods and timings has been recognized to be superior in this group or in general. As the fetus with growth restriction and/or reduced liquor volume is at particular risk of stillbirth and neonatal mortality and morbidity, ultrasonographic assessment of fetal growth and liquor volume is recommended.^43^, ^44^ Trials suggest that in high‐risk pregnancies, Doppler ultrasound of the umbilical artery may reduce perinatal death and obstetric intervention, but the evidence is not definitive^45^; it is important to note that near or at term, a normal umbilical artery Doppler does not exclude fetal compromise.^46^, ^47^, ^48^ The cerebroplacental ratio is better in the prediction of adverse outcome in small‐for‐gestational age fetuses at term.^49^

At <34 weeks in the presence of fetal growth restriction, the addition of Doppler ultrasound of the ductus venosus may be beneficial, as absent or reserved end‐diastolic velocities are associated with a substantially increased risk of stillbirth^50^; initiation of delivery for abnormal ductus venosus Doppler, short‐term fetal heart rate variation by computerized cardiotocography (cCTG), and/or spontaneous fetal heart rate decelerations is associated with improved neurodevelopmental outcomes among survivors.^51^, ^52^, ^53^, ^54^ In hypertensive pregnancy with early fetal growth restriction, we recommend against using the biophysical profile for fetal surveillance as it may be falsely reassuring and, when abnormal, is a late finding.^43^, ^55^, ^56^, ^57^ Where available, cardiotocography should be performed daily based on the 5% daily risk of abnormality seen in the TRUFFLE study.^58^

Without ready access to methods of fetal surveillance beyond fetal heart rate monitoring, maternal characteristics may identify perinatal risk. Maternal age, number of symptoms (0, 1, or ≥2), and dipstick proteinuria can be used to estimate perinatal risk at ≥32 weeks; before this time, risk is almost entirely driven by gestational age.^59^ Women at increased risk may benefit from transfer to facility‐based care, but this model requires external validation to confirm performance. With access to laboratory testing, elevated serum uric acid (particularly when gestational age‐corrected) may further identify fetuses at risk.^60^ With access to angiogenic markers, a low PLGF (<50 pg/mL) may identify fetuses at particular risk of stillbirth in low‐ and middle‐income countries.^61^

### 7.3 Nonpharmacological therapy

**Table:** 

Pragmatic practice advice	Quality of evidence	Strength of recommendation
There is insufficient evidence to recommend for or against restricted activity, in hospital or at home, for any hypertensive disorder of pregnancy.	Low 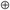 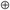 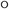 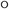	Conditional

Of note, for women with gestational hypertension, some bed rest in hospital was superior to unrestricted activity at home, but the trial was small (218 women) and performed 25 years ago.^62^ In a similar trial that examined different endpoints, women preferred unrestricted activity at home.^63^, ^64^

### 7.4 Antihypertensive therapy

**Table:** 

Best practice advice	Quality of evidence	Strength of recommendation
*Nonsevere hypertension*		
We recommend that elevated blood pressure in pregnancy be treated with antihypertensive therapy and that the target systolic blood pressure and diastolic blood pressure should be 135 and 85 mmHg, respectively.	High 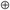 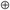 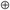 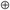	Strong
We recommend that oral labetalol, nifedipine, and methyldopa be considered as first‐line antihypertensive agents for nonsevere hypertension.	Moderate 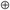 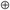 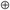 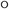	Strong
*Severe hypertension*		
We recommend that severe hypertension in pregnancy be treated urgently with antihypertensive therapy, in a monitored setting.	Moderate 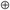 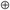 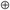 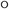	Strong
We recommend that severely elevated diastolic blood pressure be lowered to a target of 85 mmHg, but gradually over hours to days.	Low 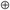 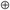 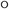 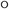	Strong
We recommend that oral nifedipine, oral labetalol, intravenous labetalol, and intravenous hydralazine be considered as first‐line antihypertensive agents for severe hypertension.	Moderate 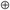 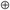 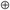 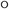	Strong

The threshold for treatment of hypertension in pregnancy is a systolic blood pressure ≥140 mmHg and/or a diastolic blood pressure ≥90 mmHg. This is true whether the hypertension is chronic, gestational, or due to pre‐eclampsia. Treatment reduces the likelihood of developing severe maternal hypertension and other complications, such as low platelets and elevated liver enzymes with symptoms based on the findings from randomized controlled trials, including the CHIPS trial.^65^, ^66^ While CHIPS enrolled women with chronic or gestational hypertension, almost half of the women developed pre‐eclampsia and all stayed on their allocated blood pressure control for an average of 2 weeks before birth. In the CHIPS trial, severe hypertension was similar to pre‐eclampsia in being a surrogate marker for adverse outcomes.^67^

**FIGURE 1:**
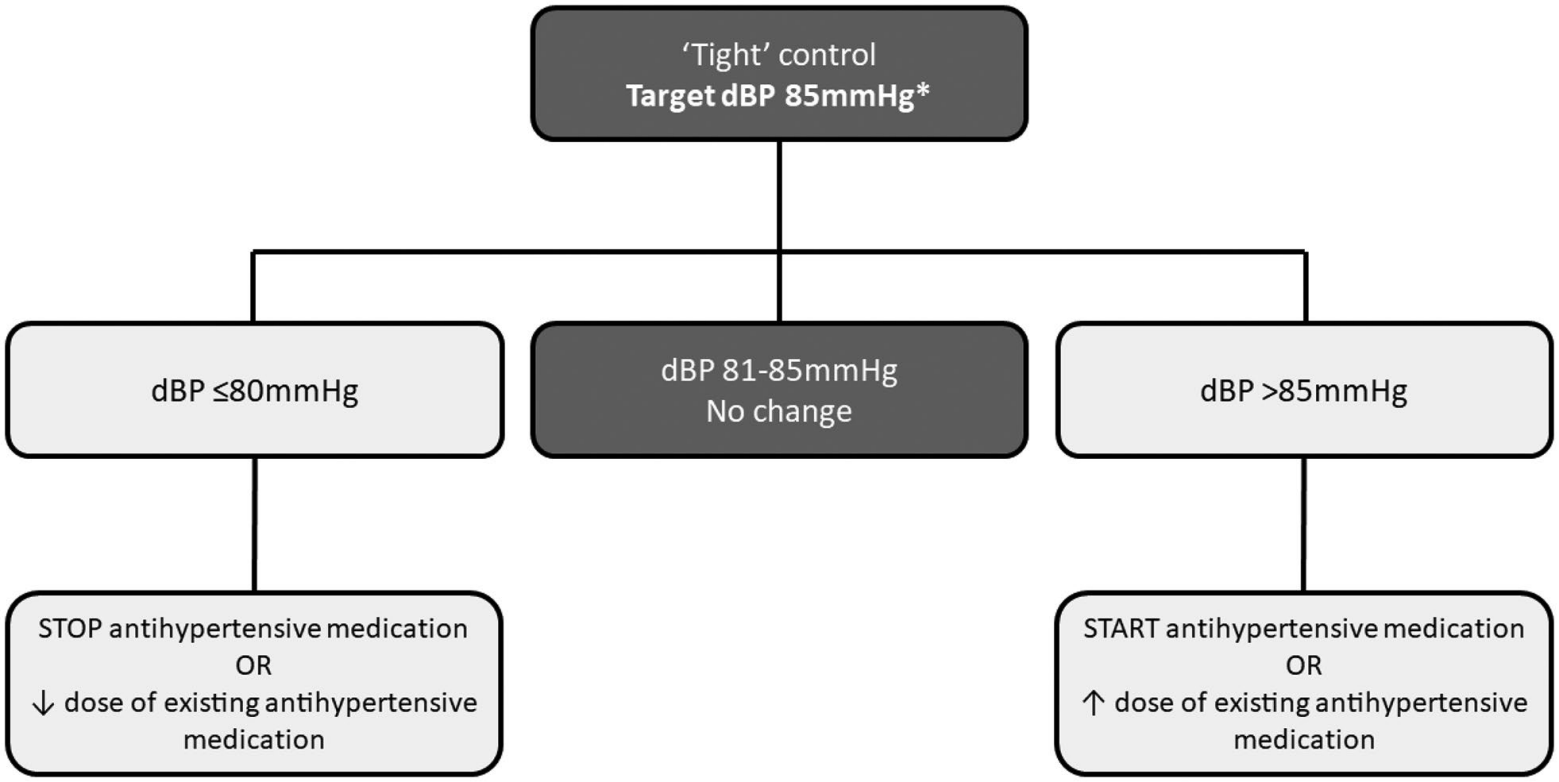
Algorithm for “tight” blood pressure control used in the CHIPS trial.^a^ *If systolic blood pressure is ≥160 mmHg, increase dose of existing medication or start new antihypertensive medication to get systolic blood pressure <160 mmHg, regardless of diastolic blood pressure (dBP). ^a^Adapted figure reprinted with permission from Wiley: Magee LA, Khalil A, von Dadelszen P. Pregnancy hypertension diagnosis and care in COVID‐19 era and beyond. *Ultrasound Obstet Gynecol*. 2020;56:7–10. © 2020 ISUOG. Permission for original figure reprinted from Pregnancy Hypertens. 2019;18. Magee LA, Rey E, Asztalos E, et al. Management of non‐severe pregnancy hypertension – a summary of the CHIPS Trial (Control of Hypertension in Pregnancy Study) research publications. 156–162. © 2019, with permission from Elsevier.

The target blood pressure for antihypertensive treatment should be a diastolic blood pressure of 85 mmHg, as in CHIPS; this approach achieved a mean blood pressure of 133/85 mmHg by use of a simple algorithm in which antihypertensive drugs were reduced or ceased if diastolic blood pressure fell below 80 mmHg and increased or started if it rose above 85 mmHg, or systolic blood pressure was ≥160 mmHg (regardless of diastolic blood pressure) (Figure 1).

The approach to hypertension is the same for all women, including those with comorbidities such as chronic renal disease.^68^ The only exception is white‐coat hypertension unless women develop blood pressure levels ≥160/110 mmHg in the office/hospital setting.

No antihypertensive agent has been shown to be superior to others for treatment of nonsevere hypertension, but oral labetalol, nifedipine, and methyldopa are used most commonly. Less commonly used but acceptable antihypertensive agents include other beta‐blockers (e.g. oxprenolol).^69^ Other potential agents are less desirable but not contraindicated, based on unproven concerns about maternal tachycardia when used alone (i.e. hydralazine), stillbirth in the setting of pre‐eclampsia (i.e. prazosin), or theoretical hazards of reduced maternal circulating volume (i.e. diuretics).

While all hypertension in pregnancy warrants antihypertensive therapy, treatment is warranted urgently when the blood pressure elevation is severe: to levels of systolic blood pressure ≥160 mmHg or diastolic blood pressure ≥110 mmHg. While there are no trials that have demonstrated that antihypertensive therapy is superior to placebo/no therapy for severe hypertension, such trials would be unethical, and there is international consensus that these women require urgent treatment to decrease the risk of intracerebral events and other complications; severe hypertension is a surrogate marker for adverse maternal and perinatal outcomes and these women require close monitoring even after blood pressure has come down.^67^ Advice to lower blood pressure gradually is based on exacerbation of cerebral ischemia in stroke and an excess of adverse perinatal outcomes among women treated with agents that lower blood pressure quickly.^70^, ^71^, ^72^

There is no antihypertensive agent that has proven to be superior to others for treatment of severe hypertension in pregnancy. A recent study showed that oral nifedipine retard use resulted in a greater frequency of primary outcome attainment (blood pressure control, defined as 120–150 mmHg systolic and 70–100 mmHg diastolic) within 6 hours with no adverse outcomes) than oral labetalol or methyldopa use.^71^ However, oral nifedipine, oral labetalol, intravenous labetalol, and intravenous hydralazine are most commonly used. Traditionally, oral nifedipine (often capsules) or parenteral antihypertensive agents have been used to treat severe hypertension, but other oral agents, such as oral labetalol (200 mg orally hourly, for three doses if necessary) or oral methyldopa (1 g as a single dose) may be effective in the majority of women.^69^, ^71^, ^73^ They are worth considering as an alternative, particularly during transfer to a monitored setting.

While oral antihypertensives can be given during labor, these are associated with reduced gastrointestinal motility and drug absorption. As such, if blood pressure control is suboptimal during labor, parenteral agents may be needed.

### 7.5 Magnesium sulfate and other strategies for women with pre‐eclampsia

**Table:** 

Best practice advice	Quality of evidence	Strength of recommendation
We recommend magnesium sulfate to prevent recurrent seizures for women with eclampsia.	High 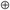 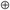 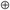 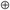	Strong
We recommend magnesium sulfate as a neuroprotective agent preventing perinatal morbidity in preterm pre‐eclampsia requiring delivery at <32 weeks.	Moderate 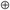 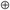 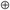 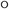	Strong
We recommend magnesium sulfate to prevent eclampsia for women with pre‐eclampsia who either have blood pressure ≥170/110 mmHg and ≥3+ proteinuria, or blood pressure ≥150/100 mmHg, ≥2+ proteinuria, and neurological signs or symptoms of “imminent eclampsia.”	High 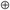 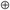 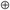 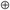	Strong
For prevention of recurrent or first seizures, magnesium sulfate should be used in standard dosage, usually a 4‐g intravenous loading dose followed by maintenance of either 5 g intramuscularly to each buttock every 4 hours or 1 g per hour intravenously, for 24 hours.	Moderate 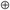 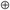 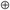 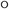	Strong
We do not recommend plasma volume expansion for women with pre‐eclampsia.	Moderate 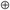 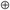 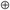 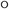	Strong

There is clear evidence that magnesium sulfate halves both the incidence of seizures among women with pre‐eclampsia, and the recurrence of seizures among women with eclampsia.^74^ Among women with pre‐eclampsia, the number needed to treat (NNT) is approximately 100 to prevent one seizure. However, it is controversial whether all women with pre‐eclampsia should receive magnesium sulfate due to an elevated risk of cesarean delivery, more maternal adverse effects, and higher costs (i.e. USD [year 2001] $23 000 to prevent one seizure if administered to all women with pre‐eclampsia).^75^ As the NNT is lower among women with “severe” pre‐eclampsia (approximately 50), it is reasonable in well‐resourced settings to restrict magnesium sulfate use to “severe” pre‐eclampsia as defined in the Magpie trial^74^: blood pressure ≥170/110 mmHg and ≥3+ proteinuria, or blood pressure ≥150/100 mmHg with ≥2+ proteinuria and neurological signs or symptoms of “imminent eclampsia” (which was not defined but is taken to mean headache, visual symptoms, or clonus). Each unit should have a consistent policy concerning their use of magnesium sulfate.

The dosing regimens used in the Magpie^74^ trial should be used (e.g. 4 g intravenous loading and 1 g per hour maintenance intravenously). This includes continuation of magnesium sulfate for 24 hours, until further evidence is published on the effectiveness of alternative dosing that is either smaller in dose or abbreviated in duration. Monitoring of serum magnesium levels is not necessary unless there is reduced kidney function or another reason for heightened risk of toxicity.

There is evidence that antenatal magnesium sulfate given prior to preterm birth for fetal neuroprotection prevents cerebral palsy and reduces the combined risk of fetal/infant death or cerebral palsy. Benefit is seen regardless of the reason for preterm birth, with similar effects across a range of preterm gestational ages and different regimens.^76^

Hypertensive pregnancy is a major cause of iatrogenic prematurity. Antenatal corticosteroids for acceleration of fetal pulmonary maturity should be used in hypertensive as in other pregnancy based on gestational age criteria and local policy.

Plasma volume expansion with colloid solutions does not improve pregnancy outcomes, and may increase the need for cesarean delivery, decrease pregnancy prolongation, and increase the risk of pulmonary edema.^77^, ^78^ For women with pre‐eclampsia, total fluid intake in labor should be restricted to approximately 80 mL per hour.^79^

### 7.6 Timed delivery

**Table:** 

Best practice advice	Quality of evidence	Strength of recommendation
We recommend delivery for women with any hypertensive disorder of pregnancy at any gestational age in the presence of one or more of the conditions listed below: abnormal neurological features such as severe intractable headache, repeated visual scotomata, eclampsia, or stroke; repeated episodes of severe hypertension despite maintenance treatment with three classes of antihypertensive agents; pulmonary edema or oxygen saturation <90%; progressive thrombocytopenia (particularly <50 × 10^9^/L or need for transfusion); abnormal and rising serum creatinine; abruption with evidence of maternal or fetal compromise; nonreassuring fetal status (including intrauterine fetal death).	Moderate 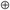 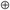 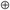 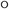	Strong
<34^+0^ weeks (very preterm):		
We suggest that at <34^+0^ weeks, expectant care be undertaken for women with chronic or gestational hypertension unless there is an indication for birth.	Very low 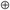 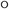 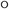 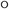	Conditional
We suggest expectant management be considered for women with pre‐eclampsia at <34^+0^ weeks, but only in tertiary centers with experience of careful noninvasive monitoring of the mother and capable of support for very preterm infants.	Moderate 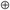 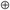 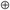 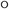	Conditional
34^+0^–36^+6^ weeks (late preterm):		
We suggest that at 34^+0^–36^+6^ weeks, expectant care be undertaken for women with chronic or gestational hypertension unless there is an indication for birth.	Very low 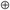 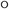 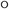 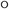	Conditional
We suggest that initiation of delivery be discussed for women with pre‐eclampsia at 34^+0^–35^+6^ weeks, as it decreases maternal but increases neonatal risk.	Moderate 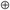 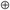 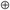 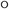	Conditional
We recommend initiation of birth for women with pre‐eclampsia at 36^+0^–36^+6^ weeks.	Moderate 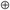 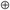 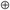 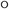	Strong
37^+0^–41^+6^ weeks (term):		
We suggest that for women with chronic or gestational hypertension, initiation of delivery be discussed at 38^+0^ to 39^+6^ weeks but should be advised from 40^+0^ weeks.	Low 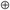 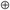 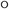 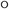	Conditional
We suggest that for women whose gestational hypertension developed preterm, initiation of delivery can be offered at 38^+0^ to 39^+6^ weeks, but should be advised by 40^+0^ weeks.	Moderate 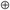 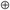 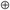 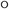	Strong
We recommend delivery be initiated within 24 hours for women with gestational hypertension or pre‐eclampsia that develops at term.	Moderate 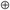 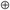 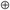 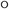	Strong

Indications for planned birth, regardless of gestational age or hypertensive disorder, include those end‐organ complications associated with a heightened risk of maternal or perinatal death.^80^ For women with pre‐eclampsia, neither the serum uric acid nor the level of proteinuria should be used as indications for delivery.

At <34^+0^ weeks there are no data to indicate that women with chronic or gestational hypertension would benefit from delivery unless there is a specific indication for birth, as listed above. At this gestational age for women with pre‐eclampsia, small randomized controlled trials suggest that expectant care may improve neonatal outcomes without increasing maternal risk.^81^ However, expectant care should be undertaken only where there are adequate services to support the needs of a sick mother and baby.

At 34^+0^–36^+6^ weeks there are few data to guide care of women with chronic or gestational hypertension. One study on timing of birth included women with chronic hypertension, but they had either superimposed pre‐eclampsia or “deteriorating hypertension” that satisfies the definition of superimposed pre‐eclampsia by many guidelines.^82^ The HYPITAT‐II trial included 182 women with gestational hypertension. While outcomes were similar to those of women with pre‐eclampsia in subgroup analyses, initiation of birth may have been associated with reduction in maternal but an increase in neonatal risk; however, the number of women randomized was insufficient on which to base a recommendation.^82^

At 34^+0^–36^+6^ weeks for women with pre‐eclampsia, randomized controlled trial data suggest that initiation of birth, which results in delivery an average of 5 days earlier than ongoing expectant care, is associated with reduced maternal morbidity and severe hypertension, but increased neonatal morbidity, particularly respiratory problems. Initiation of birth was associated with more neonatal respiratory morbidity in the Dutch HYPITAT‐II trial (703 women) in which 1% of women received antenatal steroids.^82^ On the other hand, in the PHOENIX trial (900 women), initiation of birth was associated with reduced maternal morbidity and more neonatal care unit admission, but no increase in neonatal respiratory morbidity.^83^ Although the women in the PHOENIX trial were at higher risk of adverse outcomes (based on all having pre‐eclampsia versus just under half in the HYPITAT‐II trial), a key consideration was that 60% of women in the PHOENIX trial received antenatal corticosteroids, which may explain why no difference was seen in respiratory distress in this trial.^83^ Reassuringly, however, initiation of birth (versus expectant care) has been associated with similar child development and behavior outcomes at the age of 5 years.^84^ An individual patient data meta‐analysis suggested that neonatal risk associated with initiation of birth at 34^+0^–36^+6^ weeks may be focused on the 34^+0^–35^+6^ window, with no increased risk from 36^+0^ weeks^85^; this finding is consistent with subgroup analyses in the PHOENIX trial.^83^

At term gestational age, women with chronic hypertension may benefit from birth at 38^+0^–39^+6^ weeks, in terms of reduced incidence of severe hypertension, stillbirth, and cesarean delivery, but the evidence is primarily observational in nature^86^, ^87^; randomized controlled trial data on 50 women suggest that initiation of delivery at 37^+0^ weeks is associated with an excess of neonatal morbidity.^88^ There is one ongoing trial of timed delivery at term that is including women with chronic hypertension and preterm gestational hypertension (ISRCTN77258279). Women with gestational hypertension or pre‐eclampsia that develops at term should be offered initiation of birth within 24 hours based on the results of the HYPITAT‐I trial.^89^ A meta‐analysis of the PHOENIX trial, relevant women in HYPITAT‐II, and other relevant trials is underway.

It is important to note that labor induction does not increase cesarean delivery. In fact, in pregnancy hypertension trials, labor induction at or near term has been associated with a nonsignificant reduction in cesarean delivery. In labor induction trials taken together, labor induction decreased (not increased) cesarean delivery.^90^ The PHOENIX trial was associated with significantly more spontaneous vaginal deliveries in the group routinely delivered.^83^ Furthermore, initiation of birth versus expectant care trials have been conducted in environments in which hypertension is treated when substantially elevated, such as ≥150/100^83^ or ≥160/110 mmHg,^84^, ^91^ an important fact given the key outcome of severe hypertension, which can be halved in incidence by antihypertensive therapy.^65^

### 7.7 Postpartum care

**Table:** 

Pragmatic practice advice	Quality of evidence	Strength of recommendation
Blood pressure should continue to be monitored after delivery until 6 days postpartum, as it is likely to be highest 3–6 days after birth.	Low 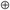 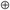 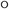 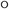	Conditional
We suggest that antihypertensive therapy that has been administered before birth be continued after birth for as long as required to maintain blood pressure control.	Low 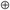 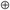 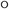 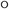	Conditional
We suggest that consideration be given to administering antihypertensive therapy for any hypertension diagnosed before 6 days postpartum.	Low 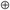 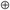 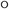 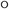	Conditional
We suggest that nonsteroidal anti‐inflammatory drugs for postpartum analgesia can be used in women with pre‐eclampsia unless blood pressure is uncontrolled, there is known renal disease, or pre‐eclampsia has been associated with placental abruption, acute kidney injury, or other known risk factors for acute kidney injury (e.g. sepsis, postpartum hemorrhage).	Low 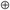 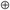 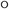 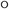	Conditional

Women may develop pre‐eclampsia or complications related to pre‐eclampsia (including eclampsia) for the first time after birth. The highest blood pressure values may occur after women leave the monitored inpatient setting, so it is important to have a blood pressure monitoring plan in place. Most antihypertensive agents, including ACE inhibitors are acceptable in breastfeeding, and up‐to‐date information can be obtained from the LactMed database (https://toxnet.nlm.nih.gov/newtoxnet/lactmed.htm).

Initial concerns that use of nonsteroidal anti‐inflammatory drugs (NSAIDs) may increase hypertensive urgency when used after birth following hypertensive pregnancy^92^ have not been confirmed. Retrospective cohort studies (involving 538 women, mostly with more advanced pre‐eclampsia) suggest that NSAIDs do not increase postpartum blood pressure, antihypertensive dose or need for dose escalation, maternal complications, readmission, or opioid use.^93^, ^94^, ^95^ Two randomized controlled trials of ibuprofen versus acetaminophen/paracetamol for postpartum analgesia for “severe” pre‐eclampsia have been reassuring, finding either no increase in hypertension to 6 weeks after birth^96^ or an increase in blood pressure but no increase in the incidence of severe hypertension.^97^ As such, NSAIDs may be used for postpartum analgesia following hypertensive pregnancy, as long as blood pressure control is not a problem and there are not other risk factors for postpartum acute kidney injury (e.g. postpartum hemorrhage or chronic kidney disease).

## 8 LONG‐TERM CONSIDERATIONS ASSOCIATED WITH PRE‐ECLAMPSIA

**Table:** 

Pragmatic practice advice	Quality of evidence	Strength of recommendation
We recommend that hypertensive pregnancy disorders should be acknowledged as predictors of long‐term maternal cardiovascular morbidity.	Moderate 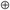 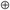 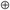 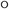	Conditional
We recommend that the following measures are implemented at 6–12 weeks after birth, and periodically thereafter, preferably yearly, following a pregnancy complicated by hypertensive disorders: history and physical examination; blood pressure measurements; consider screening for other cardiovascular risk factors and for diabetes according to additional risk factors.	Moderate 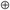 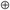 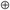 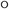	Conditional

Pre‐eclampsia is a well‐established risk factor for long‐term maternal and neonatal complications. Even after resolution of symptoms, an elevated risk for future maternal cardiovascular, cerebrovascular, and vascular disease exists.^98^, ^99^, ^100^, ^101^, ^102^, ^103^, ^104^, ^105^, ^106^, ^107^, ^108^, ^109^, ^110^ In addition, even though less investigated, several studies have already demonstrated that children antenatally exposed to pre‐eclampsia are at an increased risk of long‐term cardiovascular, respiratory neuropsychiatric, gastrointestinal, and endocrinological morbidity.^111^, ^112^, ^113^, ^114^, ^115^

### 8.1 Cardiovascular disease

Future maternal cardiovascular disease is probably the most studied long‐term consequence of hypertensive disease of pregnancy. Multiple systematic reviews of controlled studies evaluated the risk of late cardiovascular events in women with and without a history of hypertensive disease of pregnancy. In 2007, Bellamy et al.^98^ published their results of a systematic review and meta‐analysis on the risk for future cardiovascular morbidity of women who experienced pre‐eclampsia. They analyzed 25 studies including more than 3 million women, of whom about 5% had a history of pre‐eclampsia, and reported the relative risk (RR) for hypertension to be 3.70 (95% CI, 2.70–5.05), for ischemic heart disease 2.16 (95% CI, 1.86–2.52), for stroke 1.81 (95% CI, 1.45–2.27), and for venous thromboembolism 1.79 (95% CI, 1.37–2.33). In their analysis, there was also a relative risk of 1.49 (95% CI, 1.05–2.14) for overall mortality after pre‐eclampsia. Another meta‐analysis that included case–control and cohort studies found that the odds ratio for cardiac disease was 2.47 (95% CI, 1.22–5.01) in the case–control studies, and the relative risk in the cohort studies was 2.33 (95% CI, 1.95–2.78). They also reported an increased risk of cerebrovascular disease (RR 2.03; 95% CI, 1.54–2.67) and cardiovascular mortality (RR 2.29; 95% CI, 1.73–3.04). Likewise, a review of 43 studies found pre‐eclampsia to be associated with an approximate two‐fold increase in odds of cardiovascular disease and cerebrovascular disease, and a three‐fold increased risk of hypertension.^101^ In 2017, Wu et al.^100^ analyzed 22 studies with more than 6.4 million women including more than 258 000 women with pre‐eclampsia. Adjusting for potential confounders, such as age, body mass index, and diabetes mellitus, they demonstrated that pre‐eclampsia was independently associated with an increased risk of future heart failure (RR 4.19; 95% CI, 2.09–8.38), coronary heart disease (RR 2.50; 95% CI, 1.43–4.37), cardiovascular disease‐related death (RR 2.21; 95% CI, 1.83–2.66), and stroke (RR 1.81; 95% CI, 1.29–2.55), highlighting once again the importance of lifelong monitoring of cardiovascular risk factors in women with a history of pre‐eclampsia.

The strength of these data has already led the American Heart Association (AHA) in 2011 to consider a history of pre‐eclampsia or gestational hypertension a major risk factor for development of cardiovascular disease.^102^ The American College of Obstetricians and Gynecologists (ACOG), with the AHA, has published a presidential advisory with the AHA providing specific recommendations for cardiovascular disease risk factors screening for women with prior pre‐eclampsia that was preterm (<37 weeks) or recurrent.^103^ In this group of women, ACOG recommends yearly screening of blood pressure, lipids, fasting blood sugar, and body mass index. This recommendation relates only to women with preterm or recurrent pre‐eclampsia as they are at the highest risk of cardiovascular mortality; screening for women with prior term pre‐eclampsia was not addressed.

The observation made by ACOG between term and preterm pre‐eclampsia is important as the magnitude of the above findings is further emphasized by the severity, recurrence, and gestational age of onset of the hypertensive disorder.

### 8.2 Early and late onset of pre‐eclampsia

Women with early‐onset pre‐eclampsia are at a significant higher risk for vascular disease compared to late‐onset pre‐eclampsia. A Norwegian population‐based cohort study of 626 272 deliveries found that women who had pre‐eclampsia had a 1.2‐fold higher long‐term risk of death (95% CI, 1.02–1.37) than women who did not have pre‐eclampsia. When stratified by term or preterm birth, given that pre‐eclampsia might be more severe if onset is preterm, the risk increased to 2.71 (95% CI, 1.99–3.68) in women with pre‐eclampsia and preterm delivery compared to women without pre‐eclampsia who delivered at term. Furthermore, the risk of death from cardiovascular causes among women with pre‐eclampsia and preterm delivery was 8.12‐fold higher (95% CI, 4.31–15.33) than women without pre‐eclampsia who delivered at term, whereas women with pre‐eclampsia who delivered at term had only a 1.6‐fold (95% CI, 1.01–2.7) higher risk of cardiovascular death.^104^ Similar results were reported by other studies,^105^ where the hazard ratio for cardiovascular death associated with preterm pre‐eclampsia (delivery <37 weeks) was 3.7 times higher but only 1.6 times higher among women with prior term pre‐eclampsia, both compared to normotensive pregnancies.^105^

### 8.3 Severity of pre‐eclampsia

A dose–response relationship has been observed between the severity of pre‐eclampsia and the long‐term risk of cardiovascular disease. In 2015, Kessous et al.^106^ reported a significant association between pre‐eclampsia and cardiovascular morbidity and showed a linear association between the severity of pre‐eclampsia (no pre‐eclampsia, mild pre‐eclampsia, severe pre‐eclampsia, and eclampsia) and the risk of future cardiovascular morbidity (2.75% vs 4.5% vs 5.2% vs 5.7%, respectively; *P* = 0.001). Similar results were published in earlier studies^107^, ^108^ and were also found in the meta‐analysis by McDonald et al.^99^ whereby mild, moderate, and severe pre‐eclampsia were associated with relative risks of 2.00, 2.99, and 5.36, respectively, of developing future cardiovascular disease.

### 8.4 Recurrence of pre‐eclampsia

A significant linear association was documented between the number of previous pregnancies with pre‐eclampsia and the risk for future cardiovascular disease.^106^ This association was also reported in the registry‐based cohort study from Denmark,^108^ where multiparous women had a 2.8 (95% CI, 2.3–3.4) increased risk after two pregnancies complicated by pre‐eclampsia compared to a lower 1.3 (95% CI, 1.1–1.5) increased risk if only their first pregnancy was pre‐eclamptic, both compared with multiparous women without hypertensive disease. To note, the corresponding relative risks for stroke in the women in this study were 1.5 and 1.2.

### 8.5 End‐stage renal disease

Women with pre‐eclampsia may also be at increased risk of developing end‐stage renal disease (ESRD) later in life, but the absolute risk is small. A retrospective study from Norway found that women with pre‐eclampsia in their first pregnancy had a four‐fold increase in risk of ESRD compared with women without pre‐eclampsia (RR 4.7; 95% CI, 3.6–6.1), but the absolute risk of ESRD was less than 1% within 20 years.^109^ Similarly, in another study,^106^ women with pre‐eclampsia had an increased risk for renal disease later in life that was also associated with the severity of pre‐eclampsia (no pre‐eclampsia, mild pre‐eclampsia, severe pre‐eclampsia, and eclampsia) although the total prevalence was small (0.1% vs 0.2% vs 0.5% vs 1.1%, respectively; *P* = 0.001). ESRD may possibly be the sequel of a subclinical renal disease during pregnancy, but it is also possible that pre‐existing risk factors predisposed these women to both pre‐eclampsia and ESRD, just as these women are at increased risk for other cardiovascular morbidity.

### 8.6 Ophthalmic disease

The microangiopathic lesions thought to be caused by pre‐eclampsia may also expose women to long‐term ophthalmic complications such as diabetic retinopathy and retinal detachment. While investigating over 100 000 deliveries, 8.1% of them complicated with pre‐eclampsia, a recent study found that a history of pre‐eclampsia in pregnancy was independently associated with higher rates of ophthalmic morbidity that was also associated with the severity (no pre‐eclampsia, mild pre‐eclampsia, severe pre‐eclampsia, and eclampsia) of the disease (0.2% vs 0.3% vs 0.5% vs 2.2%, respectively; *P* < 0.001).^110^

## 9 CHOICE OF AUTOMATED BLOOD PRESSURE MONITORS

**Table:** 

Best practice advice	Quality of evidence	Strength of recommendation
We recommend that if automated blood pressure devices are used, only automated blood pressure devices that have been shown to be accurate in pregnancy and pre‐eclampsia should be used.	Moderate 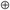 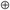 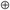 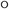	Strong

Due to the physiological cardiovascular adaptation in pregnancy, oscillometric blood pressure devices are usually inaccurate in pre‐eclampsia and tend to underestimate blood pressure. Therefore, only devices that have been shown to be accurate in measuring blood pressure in pregnancy should be relied upon. Validation will ensure both calibration and the software/hardware correctly obtains an accurate measurement.^116^ A number of validation protocols have been published, including by the British Hypertension Society, the European Society of Hypertension, and the Association for the Advancement of Medical Instrumentation. These protocols have recently been incorporated into an International Organization for Standardization standard. There are greater than 4000 devices on the market and a small number are accurate in pregnancy.^117^ Devices that have been proven valid and accurate should be used, given the consequences of inaccurate blood pressure measurement during pregnancy. Box 2 demonstrates devices that can be recommended.

Box 2 **Blood pressure devices validated for use in pregnancy and pre‐eclampsia^117^ ^a^**

**Table:** 

Hospital/clinic devices	Dinamap ProCare 400
	A&D UM‐101
	Nissei DS‐400
	Omron HEM907
	Welch Allyn QuietTrak (Ambulatory)
	BP Lab (Ambulatory)
	PAR Medizintechnik & Co. Physio‐Port (Ambulatory)
Portable devices (suitable for home use)	Omron M7 (HEM 780E)
	Omron MIT
	Omron MIT Elite
	Omron HEM‐9210T
	Omron BP760N (HEM‐7320‐Z)
	Microlife WatchBP Home A
	Microlife BP 3BTO‐A
	Microlife BP 3AS1‐2
	Microlife WatchBP Home A BT
	Microlife WatchBP Home S
	Microlife CRADLE VSA
	Andon iHealth Track

^a^ The STRIDE BP website (https://www.stridebp.org/bp‐monitors) provides an updated list of validated blood pressure monitors.

### 9.1 Blood pressure devices suitable for low‐resource settings

Mercury sphygmomanometry is no longer available. While aneroid devices are used commonly, they may over‐ or underestimate blood pressure,^118^ and they need to be regularly calibrated. Liquid‐crystal sphygmomanometery^119^ is the best alternative. Alternatively, the CRADLE VSA device (Microlife Corporation; Widnau, Switzerland) has been validated for use in pregnancy, as well as in normotensive, hypertensive, and hypotensive women, meeting the WHO’s requirements for suitability for low‐ and middle‐income countries.^120^ It is reasonably costed (USD $20), robust, easy to use, and can be portable. It does not require calibration. It can be used in both an auscultatory or oscillometric function. It has low power requirements as it is charged from a micro‐USB charger. An early warning score traffic light is triggered by raised blood pressure or an abnormal shock index (pulse:systolic blood pressure). Healthcare professionals have given unanimously positive feedback for the traffic light early warning system, and pregnant women unanimously agree.^121^ A stepped‐wedge, cluster‐randomized trial of the CRADLE VSA device in 10 clusters in eight low‐ and middle‐income countries found that introduction of the device in conjunction with an educational package resulted in no significant benefit or harm (OR 1.22; 95% CI, 0.73–2.06; *P* = 0.45) as the intercluster variation was too great to demonstrate any effect.^122^ However, a composite of maternal outcome (of death, eclampsia, and/or hysterectomy) was lower at an individual level before intervention (79.4 per 100 000 deliveries) compared with after intervention (72.8 per 100 000 deliveries).^122^ In some countries there were highly significant effects in the primary outcome, and therefore further work regarding mechanism is needed.^122^

## 10 COST‐EFFECTIVENESS OF SUPPLEMENTING CURRENT CLINICAL PRACTICE WITH PLACENTAL GROWTH FACTOR‐BASED TESTS

The diagnosis of pre‐eclampsia is based on blood pressure, maternal end‐organ involvement (i.e. proteinuria, maternal symptoms, maternal signs, and laboratory test abnormalities), and fetoplacental dysfunction. The criteria can result in false‐positive diagnoses. This may lead to unnecessary antenatal admissions, requests for multiple laboratory tests and, not infrequently, the decision of iatrogenic preterm delivery.

A Health Technology Assessment was undertaken in the UK in 2016^123^ based on three published studies^124^, ^125^, ^126^ with the aim to evaluate the diagnostic accuracy and cost‐effectiveness of PLGF‐based tests for patients referred to secondary care with suspected pre‐eclampsia at 20–37 weeks of pregnancy. The authors performed an independent economic analysis based on a decision tree model. The model evaluated costs^127^ from an NHS and Personal Social Services perspective. The total cost of managing a false‐positive diagnosis of pre‐eclampsia was £9576.25 and a true positive case of severe pre‐eclampsia was £14,545.49. Based on the modelling study, the authors concluded that the model predicts that when testing supplements routine clinical assessment to rule out and rule in pre‐eclampsia, the two tests are cost saving when performed between 20 and 35 weeks of gestation, and marginally cost saving when performed at 35–37 weeks. Length of neonatal intensive care unit stay was the most influential parameter in sensitivity analyses.

Another UK cost utility study showed that with the current clinical practice without the use of sFlt‐1/PLGF ratio test information, 36% of women were hospitalized before a diagnosis of pre‐eclampsia, of whom only 27% subsequently developed pre‐eclampsia. If the test information was available, the proportion of women hospitalized could be reduced to 16%, of whom 38% would have subsequently developed pre‐eclampsia. Among women who were not hospitalized, approximately the same proportion subsequently developed pre‐eclampsia. The introduction of the sFlt‐1/PLGF ratio is also expected to reduce the number of hospitalizations at first presentation, before developing pre‐eclampsia, from 36% to 16%.^128^ The authors concluded that the introduction of the sFlt‐1/PLGF ratio into clinical practice results in cost savings of £344 per patient compared with a non‐test (current clinical practice). Savings are primarily through an improvement in diagnostic accuracy and reduction of unnecessary hospitalization.

Independent groups from Italy^129^ and Germany^130^ similarly showed that the introduction of sFlt‐1/PLGF into hospital practice is cost saving. Savings are generated primarily through improvement in diagnostic accuracy and reduction in unnecessary hospitalization for women before the onset of pre‐eclampsia.

In a middle‐income country setting, a Brazilian group has compared the introduction of the ratio in a public and in a private hospital with expected different costs to manage patients with suspicion of pre‐eclampsia.^131^ Introduction of the sFlt‐1/PLGF ratio test resulted in cost savings in both settings: public R$185.06 and private R$635.84 per patient compared to a scenario of non‐test (current clinical practice). As expected, savings were generated primarily through reduction in unnecessary hospitalization.^131^ Currently, there are no health economic data on supplementing current clinical practice with PLGF‐based tests in low‐ and lower middle‐income countries.

The implementation of angiogenic markers in clinical practice seems to improve clinical decisions regarding hospitalization, identifying pregnant women with suspected pre‐eclampsia who are at low risk of developing the disease and thus avoiding unnecessary procedures and thus cost saving. More complicated economic analysis looking at health system opportunity costs of unnecessary hospitalization for suspected pre‐eclampsia in overburdened public services at the cost of patients with other serious but less threatening conditions is not available, but will likely show improved cost benefit of supplementing current practice with PLGF‐based testing. Predictive tools to improve clinical decision‐making are not only important for individualizing management plans to improve outcomes, but also have economic consequences for individuals, health systems, and society, and the cost‐effectiveness and cost utility of improved predictive tools are required to ensure their optimal use.

## 11 CONSIDERATIONS FOR UNIVERSAL ASPIRIN IN PRE‐ECLAMPSIA PREVENTION

Considering the clear benefit of aspirin in reducing the risk of preterm pre‐eclampsia, its low cost, and safety profile, some investigators advocate for universal aspirin prophylaxis for pre‐eclampsia prevention. It has been suggested that this would be a more cost‐effective strategy compared to using aspirin prophylaxis in women determined to be at high risk through a process of screening, which has been considered rather complex for implementation.^132^, ^133^, ^134^, ^135^ Nevertheless, possible benefits of a preventive strategy need to be balanced with potential harm due to hemorrhagic and other adverse events.^136^ Benefits of universal aspirin and long‐term safety of this strategy have not been adequately studied in randomized trials. Additionally, good adherence to treatment is paramount to successful prevention.^137^ Compliance is likely to be lower when aspirin is given to the whole population than when recommended to a selected high‐risk group of women counselled based on individual risk.^138^ Earlier trials in which pregnant women received aspirin on the sole basis of being pregnant or nulliparous demonstrated an increased frequency of bleeding episodes, low compliance with aspirin at only about 50%, and no reduction in the incidence of pre‐eclampsia.^139^, ^140^ Analogously, universal aspirin for primary prevention of cardiovascular events in healthy older adults resulted in a significantly higher risk of major hemorrhage but did not significantly reduce the risk of cardiovascular disease.^141^

## 12 RESEARCH PRIORITIES

There are three main objectives for further research. Firstly, more prospective research is required to develop and evaluate risk stratification strategies in asymptomatic unselected women. Existing evidence on the use of multimarker algorithms is promising^142^, ^143^, ^144^, ^145^, ^146^, ^147^, ^148^, ^149^, ^150^ and therefore such models require validation in other settings. Secondly, evidence of the PLGF or sFlt‐1/PLGF ratio published to date makes it highly likely that the decision when to deliver women with gestational hypertension or early disease of pre‐eclampsia after 34^+0^ weeks of gestation can be refined when these markers are added to clinical decision‐making. To date, the HYPITAT‐I and II and PHOENIX randomized controlled trials are paramount on when to deliver women with nonsevere, late‐onset hypertensive disease.^82^, ^83^, ^91^ The HYPITAT‐I trial has shown that there is no benefit to either the mother or child in prolonging pregnancy after 37 weeks of gestation in women with gestational hypertensive disease.^89^ The PHOENIX trial suggests delivery will reduce maternal morbidity.^83^ There is a need for a meta‐analysis of the smaller studies, such as the HYPITAT‐II trial, to ascertain the effects on neonatal morbidity, mainly respiratory distress syndrome. These findings must be re‐evaluated after adding knowledge from the PLGF or sFlt‐1/PLGF ratio studies.

Thirdly, the role of the PLGF or sFlt‐1/PLGF ratio to prevent fetal and/or maternal adverse events in early‐onset disease must be evaluated. The PARROT trial suggests maternal morbidity can be reduced in women with suspected disease.^26^ Although such a randomized controlled trial is hard to pull through elsewhere, a PLGF or sFlt‐1/PLGF ratio cutoff for delivery in severe early‐onset disease must be evaluated. It has been shown previously in a case–control study that the remaining pregnancy duration in women with pre‐eclampsia and an sFlt‐1/PLGF ratio of greater than 655.2 is significantly reduced. After 48 hours, only 29.4% (95% CI, 14.1–61.4%, *P* < 0.016) of the women continued their pregnancy; only 5.9% (95% CI, 0.9–39.4%) of the pre‐eclampsia/HELLP patients with an sFlt‐1/PLGF ratio above 655.2 continued their pregnancy for 7 days compared with 30.8% (95% CI, 20.5–46.3%) below this level.^151^ Therefore, these values and their ability to reduce maternal and/or fetal morbidity and mortality should be evaluated in a prospective, randomized design.

The studies presented here demonstrate that these different risk stratification strategies may show clinical value in predicting pre‐eclampsia during the second and third trimester of pregnancy. However, prospective randomized controlled trials are needed to demonstrate improvement in maternal and neonatal outcomes, in high‐risk but also in low‐risk populations.

## CONTRIBUTORS

The authors acknowledge the important contributions provided by the international expert members of FIGO’s Pregnancy and Non‐Communicable Diseases Committee during the creation of the document. Special thanks, for FIGO guidance and coordination, go to President Carlos Fuchtner, President‐Elect Jeanne Conry, and Chief Executive Mary Ann Lumsden. Special thanks also to Rachel Gooden, FIGO Project Manager.

## CONFLICTS OF INTEREST

Relating to the submitted work, Liona Poon reports receipt of a Health and Medical Research Fund grant to assess the role of pro‐ and antiangiogenic markers for pre‐eclampsia in the second and third trimester (Project No: 136168059), and a grant from Roche Diagnostics for the Asia PROGNOSIS study. Outside the submitted work, LP reports honoraria for lectures from Roche Diagnostics, Ferring Pharmaceuticals, and GE Healthcare; nonfinancial support of in‐kind contributions for research from Roche Diagnostics, PerkinElmer Inc., Thermo Fisher Scientific, and GE Healthcare, and personal fees for consultancy from Roche Diagnostics and Ferring Pharmaceuticals. Outside the submitted work, Stefan Verlohren reports personal fees from Roche Diagnostics, Thermo Fisher Scientific, and Alexion. Outside the submitted work, Fabricio da Silva Costa reports research support from PerkinElmer Inc., and personal fees from Roche Diagnostics and Thermo Fisher Scientific. Outside the submitted work, Kypros H. Nicolaides reports being Director of the Fetal Medicine Foundation. All other authors report no actual or potential conflicts of interest.
